# A Comparison of In-flight and Ground-Based Emergency Medical Events on the Clinical Demand for Outreach Medical Services at Taoyuan International Airport, Taiwan

**DOI:** 10.3389/fpubh.2021.663108

**Published:** 2021-07-23

**Authors:** Chin-Hsiang Lo, Yu-Feng Shiao, Shih-Tien Hsu

**Affiliations:** ^1^Division of Family Medicine, Department of Community Medicine, Landseed International Hospital, Taoyuan, Taiwan; ^2^Landseed Medical Clinic at Taiwan Taoyuan International Airport, Taoyuan, Taiwan; ^3^Division of Pulmonology, Department of Internal Medicine, Landseed International Hospital, Taoyuan, Taiwan; ^4^Division of Occupational Medicine, Department of Community Medicine, Landseed International Hospital, Taoyuan, Taiwan

**Keywords:** airplane travel, airport, clinical demand, disease distribution, emergency medical events, medical outreach services

## Abstract

**Background:** Limited information is available covering all medical events managed by the airport-based outreach medical service. This study explores the clinical demand for emergency medical outreach services at Taoyuan International Airport (TIA), Taiwan.

**Methods:** Electronic medical records collected from TIA medical outreach services from 2017 to 2018, included passengers' profiles, flight information, events location, chief complaints, diagnosis (using ICD-9 -CM codes), and management outcomes. Medical events distribution was stratified by location and ages, and were compared statistically.

**Results:** Among 1,501 eligible records, there were 81.8% ground-based emergency medical events (GBME), 16.9% in-flight medical events (IFME) managed after scheduled landing, and 1.3% IFME leading to unscheduled diversion or re-entry to TIA. The top three GBME diagnoses were associated with neurological (23.3%), gastrointestinal (21.2%), and trauma-related (19.3%) conditions. The top three IFME diagnosis that prompted unscheduled landings via flight diversion or re-entry were neurological (47.4%), psychological (15.8%), and cardiovascular (10.5%). The chief complaints that prompted unscheduled landings were mostly related to neurological (42.1%), cardiovascular (26.3%), and out-of-hospital cardiac arrest (OHCA) (10.5%) symptoms. A higher frequency of IFME events due to dermatologic causes in patients aged ≤ 18 years compared with adults and older adults (19 vs. 1.5% and 0, respectively); and a higher frequency of IFME due to cardiovascular causes in adults ≥ 65 years compared with patients aged ≤ 65 (15.1 vs. 9%). Among all IFME patients, six out-of-hospital deaths occurred among passengers from scheduled landings and two deaths occurred among 18 IFME passengers who were transferred to local hospitals from flight diversion or re-entry. A statistically significant difference in outcomes and short-term follow-up status was found between patients with IFME and those with GBME (*p* < 0.001).

**Conclusion:** Ground-based emergency medical events exceeded in-flight medical events at TIA. The most frequent events were related to neurological, gastrointestinal symptoms, or trauma. Results of this study may provide useful information for training medical outreach staff and preparing medical supplies to meet the clinical demand for airport medical outreach services.

## Introduction

Air travel has increased sharply worldwide in recent years with 4 billion passengers traveling on commercial airlines annually as reported in 2017 (1), and continued to rise, exceeding 4.3 billion journeys in 2018 (2). During this period, record-high global demand growth of 8% in 2017 and 7.4% in 2018 was reported for international air passenger services based on revenue passenger kilometers (RPK).

In Taiwan, due to its geographical location and increased interactions with neighboring countries, the number of airline passengers passing through the five Taiwanese airports has risen remarkably. The Taiwan Civil Aeronautics Administration (http://caa.gov.tw) reported that Taiwanese airports handled 65.9 million passengers in 2017, increased by 87% over 2008. Both the in-flight environment and travel itself are stressful for passengers physiologically and may trigger medical events in flight or after landing. The frequency of in-flight medical emergencies (IFME) is estimated to be 24 to 130 events per 1 million passengers (3, 4), and several studies have characterized the clinical spectrum of these medical events (4–7). However, limited information is available on ground-based emergency medical events (GBME) occurring within airport premises. Also, the incidence and types of emergency medical events in older adults and children traveling by air have not been thoroughly investigated. In order to prepare for the increasing demand for emergency medical services associated with air travel, the clinical spectrum and distribution of medical emergencies occurring among commercial airline passengers need further characterization.

Taoyuan International Airport (TIA) opened in February 1979 about 40 kilometers from Taipei, Taiwan's capital city. The airport covers an area of 1,223 hectares, and air traffic volume at TIA has risen steeply in recent years, with the annual number of commercial flight passengers reaching 40 million in 2016 (8, 9). The Taiwan Landseed International Hospital Medical Clinic at TIA has been run since 2002 and offers 24-h general and emergency medical services and outreach services around the airport region 365 days per year. There are two airport clinics in TIA, one for each of the two terminals. Each clinic has a physician, a nurse, and an emergency medical technician, a distinct advantage over most other airports that rely instead on nearby hospitals. Equipment and medications in the emergency medical kit for the outreach services is provided as Supplementary Table 1.

A full range of medical complaints have been reported for GBME and IFME, including emergency events associated with gastrointestinal, respiratory, and cardiovascular symptoms or existing diseases, and life-threating events such as heart attack, cardiac arrest and stroke (6, 7, 10). The types of management required for these medical events varies according to where and when they occur. Events occurring during flight may be treated on-board the aircraft if medical personnel and or equipment are present, but are more likely to be treated by emergency medical personnel on the ground after landing (6, 10). Pediatric emergencies are almost always treated in-flight although additional care may be provided after landing (11). Although medical in-flight events are reported to occur about once in every 40 commercial flights, actual emergency events occur about 1 in every 150 flights, and relatively few flights are diverted to other airports compared to on the ground (5). Decisions to divert are based on the type and severity of the event (treatable or not), remaining time of the flight, distance to the destination vs. nearest airport, and availability of emergency medical services in-flight vs. on the ground (6, 10, 12). If passengers present with a shockable rhythm that can be addressed with on-board cardiopulmonary resuscitation, the flights may not be diverted (12). The management of IFME and GBME is complex and multi-faceted, and detailed data on the incidence, causes and outcomes of flight-related medical emergencies remain limited (13).

Besides the advantage of having a specialized medical center available at the airport for emergency medical outreach services, little is known about the clinical demand and types of services provided at TIA and how and where they are executed. This study sought to explore the clinical spectrum and distribution of emergency medical services provided at TIA. For this purpose, we focused on data in 2017 and 2018, during which there was a high demand for air passenger services globally.

## Methods

### Data Collection

This study retrieved and analyzed emergency medical records of the TIA medical clinic operated by the airport-based Taiwan Landseed Hospital from January 1, 2017 through December 31, 2018. Emergency medical records are the collective reports of patients who needed emergency medical services but were unable to reach the airport clinic by themselves. In such cases, a medical team is dispatched to wherever they are needed in the airport. Individual medical records include information such as when and where the event occurred, chief complaints, diagnosis given according to the International Classification of Diseases, Ninth Revision, Clinical Modification (ICD-9-CM) codes, management outcome, and basic demographic profile of the patients.

For the purpose of analysis, chief complaints or symptoms were grouped into the following broad disease categories: Neurological (headache, motion sickness, dizziness vertigo, syncope, seizure, conscious disturbance, limb weakness); Cardiovascular (mild: palpitation or chest pain, hypertension; severe: acute myocardial infarction, arrhythmia, shock); Gastrointestinal (abdominal pain, vomiting, diarrhea, nausea, bloody stool or black stool); Respiratory (dyspnea, short of breath, respiratory infection (URI pneumonia), asthma, COPD); Psychological (nervous, anxious); Trauma-related injury (abrasion or sprain, laceration, contusion, head injury, fall); Dermatological (itchy, swelling, erythematous); Diabetes mellitus (Hyperglycemia, hypoglycemia); Genitourinary (difficulty of urination, flank pain, dysuria, urine frequency); Alcohol/Drug (overuse); Fever (undetermined/unknown cause); Musculoskeletal (musculoskeletal pain); Gynecology (dysmenorrhea, vaginal bleeding).

### Statistical Analysis

Categorical data, including sex, nationality, medical event location, chief complaints, diagnoses, and follow-up status are presented as numbers and percentages. Continuous data such as patients' ages are expressed as mean ± standard deviation (mean ± SD) with range (minimum to maximum). The percentages of symptoms of chief complaints and diagnoses are depicted in bar-graphs. Subgroup analysis stratified by event situation and age (i.e., <18, 18–64, and ≥ 65 years-) was performed. Event situations were designated as ground-based, in-flight requiring diversion or re-entry, or in-flight with scheduled landing. The statistical significance of differences between subgroups were analyzed using two-sample *t*-test for continuous variables and Pearson's Chi-square test or Fisher's exact test for categorical variables. All statistical analyses were two-tailed and performed using IBM SPSS statistical software version 22 for Windows (IBM Corp., Armonk, New York, USA). A *p*-value < 0.05 was considered statistically significant.

## Results

### Demographic Data

**Figure 1:**
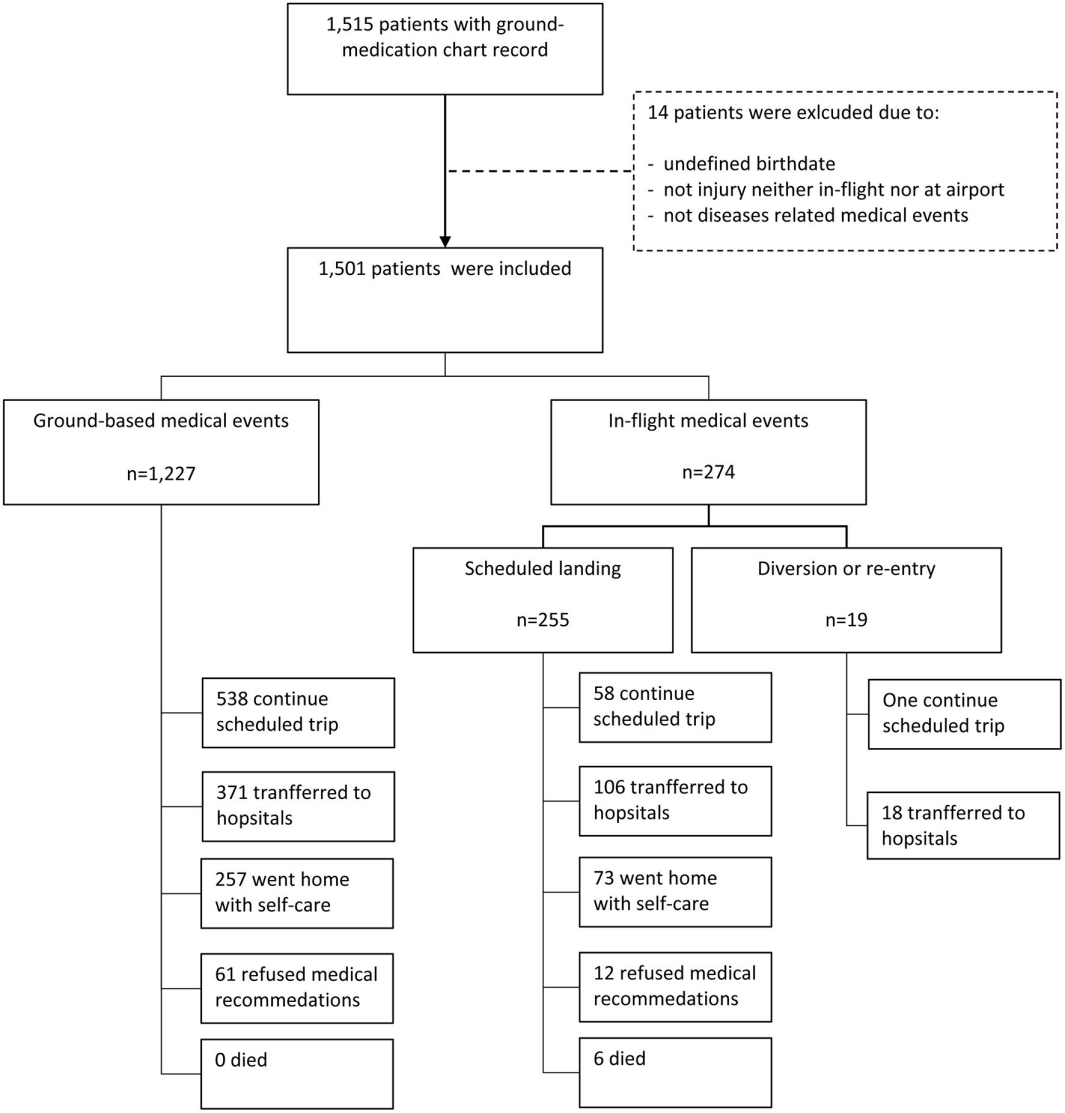
Flow-chart of patient enrollment.

A total of 1,515 individuals received outreach emergency medical services in TIA clinic during 2017/2018. Among all cases, 6% were non-passengers (i.e., flight attendants, ground crew, or other airport service personnel). Medical records with undefined birthdate, or events unrelated to emergency medical events (e.g., recent vaccination) were excluded (*n* = 14). Medical records of 1,501 patients comprising 1227 (81.7%) GBME and 274 (18.3%) IFME were included for analysis. Within IFME, 19 events resulted in unscheduled landings (13 diverted destination and 6 re-entries before take-off), and the remaining 255 events were scheduled landings (Figure 1). Patients' demographic characteristics are summarized in Table 1. Mean age of the total cohort was 43.4 ± 22.0 years. Distribution of age and sex were similar between IFME and GBME (both *p* > 0.05). The majority of patients were Taiwanese nationals (31.4%), 22.1% were US nationals, 17.9% were nationals of Southeast Asian countries, and the remaining were from other countries.

**Table 1:** Patients' characteristics at end of study period.

			**IFME**		
**Variables**	**Total** **(*N* = 1,501)**	**GBME** **(*n* = 1,227)**	**All IFME** **(*n* = 274)**	**Diversion or** **re-entry** **(*n* = 19)**	**Scheduled** **landing** **(*n* = 255)**
**Age**, years	43.4 ± 22.0 (0.22 to 94.2)	43.0 ± 22.2 (0.22 to 94.2)	45.1 ± 20.7 (0.76 to 92.8)	45.6 ± 22.2 (5.7 to 85.5)	45.1 ± 20.6 (0.76 to 92.8)
**Sex**					
Male	661 (44)	535 (43.6)	126 (46)	10 (52.6)	116 (45.5)
Female	840 (56)	692 (56.4)	148 (54)	9 (47.4)	139 (54.5)
**Nationality**					
Taiwan	472 (31.4)	366 (29.8)	106 (38.7)	4 (21.1)	102 (40)
United States	331 (22.1)	289 (23.6)	42 (15.3)	0 (0)	42 (16.5)
South-East countries	269 (17.9)	223 (18.2)	46 (16.8)	5 (26.3)	41 (16.1)
China	77 (5.1)	68 (5.5)	9 (3.3)	1 (5.3)	8 (3.1)
Canada	77 (5.1)	64 (5.2)	13 (4.7)	0 (0)	13 (5.1)
Japan	67 (4.5)	53 (4.3)	14 (5.1)	2 (10.5)	12 (4.7)
Hong Kong/Macao	57 (3.8)	50 (4.1)	7 (2.6)	3 (15.8)	4 (1.6)
Korea	35 (2.3)	24 (2)	11 (4)	3 (15.8)	8 (3.1)
Western Europe	38 (2.5)	30 (2.4)	8 (2.9)	1 (5.3)	7 (2.7)
Australia/New Zealand	25 (1.7)	20 (1.6)	5 (1.8)	0 (0)	5 (2)
India	15 (1)	14 (1.1)	1 (0.4)	0 (0)	1 (0.4)
Africa	2 (0.1)	2 (0.2)	0 (0)	0 (0)	0 (0)
Northern Europe	12 (0.8)	10 (0.8)	2 (0.7)	0 (0)	2 (0.8)
Russia/ Eastern Europe	10 (0.7)	5 (0.4)	5 (1.8)	0 (0)	5 (2)
Central and South America	10 (0.7)	7 (0.6)	3 (1.1)	0 (0)	3 (1.2)
Middle East countries	4 (0.3)	2 (0.2)	2 (0.7)	0 (0)	2 (0.8)
**Event location**					
Boarding or arrival gates	868 (57.8)	665 (54.2)	203 (74.1)	1 (5.3)	202 (79.2)
Departure or arrival Lobby and aisles (including shops and restaurants)	388 (25.8)	385 (31.4)	3 (1.1)	1 (5.3)	2 (0.8)
Baggage claim	13 (0.9)	13 (1.1)	0 (0)	-	-
In aircraft	113 (7.5)	52 (4.2)	61 (22.3)	13 (68.4)	48 (18.8)
Customs	3 (0.2)	3 (0.2)	0 (0)	-	-
Runway	14 (0.9)	7 (0.6)	7 (2.6)	4 (21.1)	3 (1.2)
Security check	71 (4.7)	71 (5.8)	0 (0)	-	-
Surrounding airport premises (including transit hotel)	29 (1.9)	29 (2.4)	0 (0)	-	-
Transfer counter	2 (0.1)	2 (0.2)	0 (0)	-	-

*Data are summarized as n (%) for age strata, sex, nationality, and event location; and age was summarized as mean ± standard deviation (SD) with range (min. to max.)*.

*No significance was derived between IFME and GBME or between IFME with diversion /or re-entry and scheduled landing*.

*GBME, ground-based medical events; IFME, in-flight medical events*.

Among all patients, the top five most frequently encountered chief complaints and diagnoses were associated with neurological, gastrointestinal, trauma, respiratory or cardiovascular symptoms (Supplementary Figure 1).

### Comparison Between In-Flight Medical Events vs. Ground-Based Medical Events

**Figure 2:**
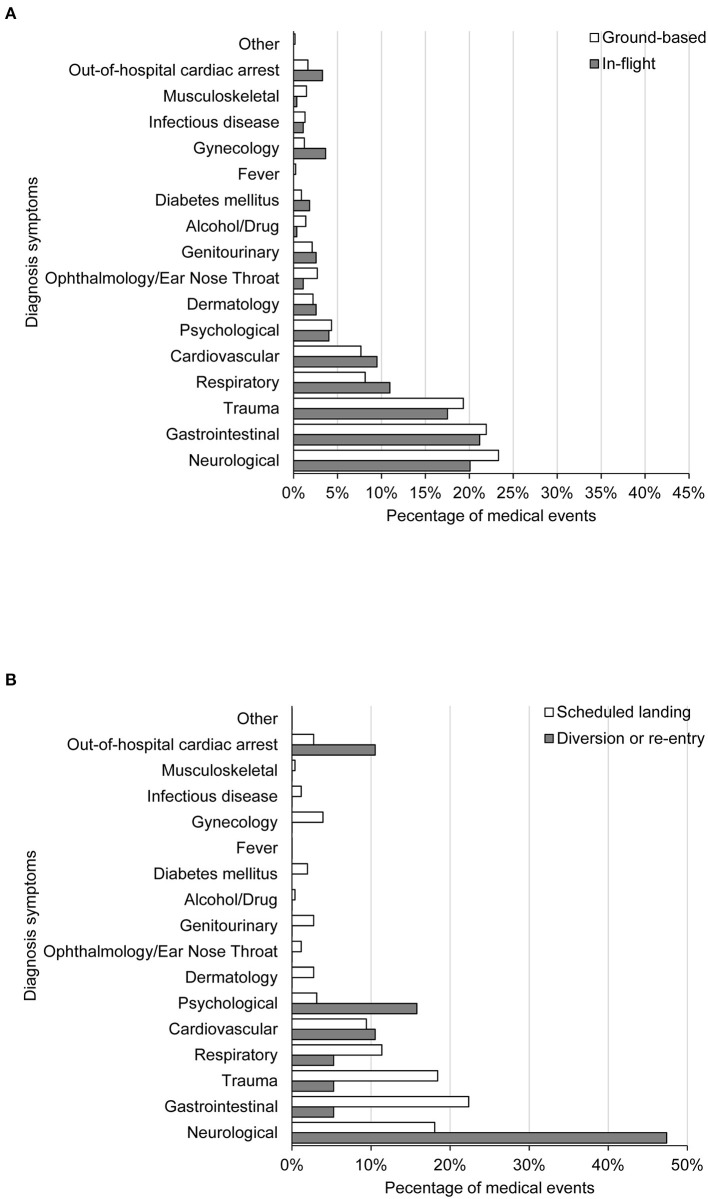
Disease symptoms by diagnosis between in-flight and ground-based medical events **(A)** and between in-flight medical events with diversion or re-entry and scheduled landing **(B)**.

Figure 2A shows the symptoms of diagnosis between IFME and GBME cases. The top five diagnoses of GBME were associated with neurological (23.3%), gastrointestinal (21.2%), trauma-related (19.3%), respiratory (8.1%), or cardiovascular (7.7%) events. The top five diagnoses of IFME were associated with gastrointestinal (21.2%), neurological (20.1%), trauma-related (17.5%), respiratory (11.0%), or cardiovascular (9.5%) events.

The three most frequent symptoms of chief complaints of GBME cases and IFME were associated with neurological, gastrointestinal, trauma-related (Supplementary Figure 2A). Differences in distribution of chief complaints between the two types of medical emergencies were statistically significant (*p* = 0.012).

### Comparison of In-Flight Medical Events With Scheduled Landings and Those That Prompted Unscheduled Landings (i.e., Diversion or Re-Entry)

The top 5 diagnoses given for IFME with scheduled landings were related to gastrointestinal (22.4%), trauma-related (18.4%), neurological (18%), respiratory (11.4%), or cardiovascular (9.4%) events (Figure 2B). The most common diagnoses that prompted unscheduled landings (flight diversion or re-entry) were related to neurological (47.4%), psychological (15.8%), cardiovascular (10.5%) conditions. There were two out-of-hospital cardiac arrests (OHCA) (10.5%) that required unscheduled landings. Differences in diagnoses between the two types of IFME were statistically significant (*p* = 0.043).

The top chief complaints of IFME in flights that made scheduled landings were related to gastrointestinal, neurological, trauma (Supplementary Figure 2B). The chief complaints that prompted unscheduled landings (*n* = 19) were related to neurological (8), cardiovascular (5), OHCA (2), respiratory (2), gastrointestinal (1), and trauma (1) symptoms/causes, in order of frequency (Table 2). Differences in chief complaints between the two types of IFME were statistically significant (*p* = 0.045).

**Table 2:** Chief complaints requiring unscheduled emergency landing, including flight-diversion/ re-entry, and final diagnosis and treatment provided.

	**Chief complaints**	**Diagnosis**	**Received treatment or management for**
	**(*n* = 19)**	**(*n* = 19)**	
Neurological	8	9	IVH; epilepsy; cerebrovascular accident (stroke), brain CT: mild dilated ventricle; syncope; hypoglycemia; seizure; conscious disturbance; motion sickness
Cardiovascular	5	2	Non-ST elevation myocardial infarction; angina pectoris unspecified
OHCA	2	2	
Gastrointestinal complaints	1	1	Acute vascular disorder of intestine
Trauma	1	1	Herniation of intervertebral disc
Respiratory	2	1	Asthma
Psychological	0	3	Psychiatric panic; hyperventilation

*IVH, intraventricular hemorrhage; OHCA, out-of-hospital cardiac arrest*.

### Comparison of Diagnoses Between In-Flight and Ground-Based Medical Events Stratified by Age

In patients aged ≤ 18 years, the top three GBME diagnoses were respiratory disease (26.7%), gastrointestinal disease (24.6%), and trauma (21.9%) (Supplementary Table 2). The top IFME diagnoses were gastrointestinal disease (23.8%), respiratory disease (23.8%), neurological diseases (19%), and dermatologic disease (19%). In adults aged between 18 and 64 years, the top three GBME and IFME diagnoses were neurological disease, gastrointestinal disease, and trauma, and among older adults (age ≥ 65 years), the top three diagnoses of both GBME and IFME were neurological disease, trauma, and cardiovascular diseases.

Among the 19 IFME that resulted in flight diversion or re-entry, only 2 occurred in children (1 neurological and 1 respiratory disease), and 2 in older adults (1 neurological and 1 OHCA) (Supplementary Table 2).

### Follow-Up Status After Medical Recommendations

Table 3 summarizes patients' outcomes and short-term follow-up. Among 1,227 individuals with GBME, 43.8% continued their scheduled trips, 30.2% were transferred to local hospitals, 20.9% were discharged with self-care, and 5% refused medical recommendations.

**Table 3:** Medical recommendation and follow-up given for ground-based or in-flight medical events.

	**IFME**		**GBME**
**Follow-up status**	**Diversion or re-entry** **(*n* = 19)**	**Scheduled landing** **(*n* = 255)**	**(*n* = 1,227)**
Continue scheduled trip	1 (5.3)^a^	59 (23.1)	538 (43.8)
Go home with self-care	0	75 (29.4)	257 (20.9)
Refused medical recommendation	0	12 (4.7)	61 (5)
Died	0	6 (2.4)	0
Transfer to local hospitals	18 (94.7)	106 (41.6)	371 (30.2)
ER then discharged	8^b^	48	201
AMA discharge from ER	0	3	12
Died during ER	1^c^	0	13
Hospitalized or transferred to other hospitals	7^d^	37	74
AMA discharge during hospitalization	1^e^	2	1
Died during hospitalization	1^f^	1	1
Lost to follow-up	0	15	69

*Statistical significance (p < 0.05) was derived for comparison of follow-up status between ground-based or in-flight medical events*.

a,b,c,d,e,f *Diagnoses for last follow-up between 19 IFME cases with diversion or re-entry, including ^a^one (neurological) for continue traveling, ^b^eight (2 cardiovascular, 2 neurological, 3 psychological, 1 respiratory) for ER then discharged, ^c^one (OHCA) who died during ER, ^d^6 (1 gastrointestinal, 6 neurological) for hospitalized or transferred to other hospitals, ^e^one (trauma) AMA discharge during hospitalization, and ^f^one (OHCA) who died during hospitalization*.

*IFME, in-flight medical events; GBME, ground-based medical events; AMA, against-medical-advice discharge; ER, emergency room*.

Among 274 patients with IFME, 21.9% continued their scheduled trips (including 1/19 case from unscheduled landings), 45.3% transferred to local hospitals (including 18/19 cases from unscheduled landings), 27.4% discharged with self-care, 4.4% refused medical recommendations, and 2.2% were pronounced dead prior to medical recommendations (Table 3). Among 124 patients transferred to local hospitals for IFME, 56 patients were discharged after receiving care in the ER, 44 were hospitalized or transferred to other hospitals, 3 were discharged AMA (against medical advice) from the ER, 3 were discharged AMA after hospitalization, 2 died after hospitalization, 1 died in the ER (from unscheduled landing), and the remaining patients were lost to follow-up.

A statistically significant difference in outcomes was found between patients with IFME and those with GBME (*p* < 0.001).

Among those 19 IFME with diversion or re-entry cases, two travelers diagnosed as OHCA (1 in ER, 1 hospitalized) died; eight cases diagnosed as cardiovascular (*n* = 2), neurological (*n* = 2), psychological (*n* = 3), and respiratory (*n* = 1) were transferred to ER then discharged; seven cases diagnosed as gastrointestinal (*n* = 1), and neurological (*n* = 6) were hospitalized or transferred to other hospitals; one diagnosed with trauma was discharged as AMA during hospitalization; and the remaining case who continued traveling was diagnosed as neurological disease.

## Discussion

In the present study, while the diagnoses given for medical emergencies were not significantly different between IFME and GBME, a statistically significant difference was found in outcomes between patients with IFME and those with GBME, as well as in their initial chief complaints. This is reflected in the diagnoses given for medical events that prompted unscheduled landings, which were significantly different from the medical events in flights that landed according to schedule, and usually more severe. IFME represented 18.3% of all cases of medical emergency services provided at the TIA medical clinic, and 19 of those events prompted flight diversion or re-entry. In comparison to IFME, a higher proportion of GBME patients continued their scheduled trips and a lower proportion were transferred to hospitals or died from the emergency event.

Almost 90% of the medical emergencies analyzed in the present study were due to neurological, gastrointestinal, traumatic, respiratory, or cardiovascular causes, which coincided largely with medical emergency cases reported by other authors in the literature (Table 4) (3–7, 11, 13–26). Peterson et al. (7) reported similar findings, concluding that most IFME were related to respiratory or gastrointestinal symptoms and patients were treated by a physician on board or in hospitals after landing. Epstein et al. (5) reported that the majority of medical events were non-life-threatening and loss of consciousness was most common, followed by cardiovascular emergencies; emergencies requiring flight diversion were rare, as were in-flight deaths. Although no differences were found in the clinical spectrum between in-flight and ground-based emergencies in the present study, differences were found in the severity of the two types of events judging by the outcomes of hospitalization and death. Previous studies commented primarily on in-flight events, and few investigated medical events occurring on airport premises.

**Table 4:** Summary of in-flight and ground-based emergency medical events reported in the literature.

**References**	**Study title**	**Number of patients/events**	**In-flight medical emergency events (Top 5)**
Al-Zurba et al. (14)	Medical problems encountered among travelers in Bahrain international airport clinic	3,350	(Airport Clinic), Upper respiratory tract (24.4%), Headache (19.2%), Musculoskeletal (12.9%), Gastroenteritis (11.0%), Medication and assist (6.9%)
Alonso-Canovas et al. (15)	Neurology at the airport	In-flight: 31 Ground: 44	In-flight Neurology events: Total (41.3%), Seizures (38.5%), Stoke (27.8%) Ground-based Neurology events: Total (58.7%), Seizures (59%), Stoke (72.2%)
Chan et al. (16)	Medical emergencies at a major international airport: In-flight symptoms and ground-based follow-up	742	Syncope (28.0%), Nausea and/or Diarrhea (12.9%), Abdominal Pain/ GU (10.3%), Chest Pain (10.7%), Behavioral/Miscellaneous (9.4%)
Cocks and Liew (17)	Commercial aviation in-flight emergencies and the physician.	Review	Common Diseases: Neurological, Cardiac, Respiratory, Gastrointestinal
Cummins and Schubach (18)	Frequency and types of medical emergencies among commercial air travelers	In-flight: 180 Ground: 559	*In-flight:* Gastrointestinal (15%), Cardiac related (20%), Trauma (14%); Respiratory (8%), Seizures (6%) *Ground-based:* Gastrointestinal (11%), Cardiac related (7%), Trauma (9%), Respiratory (7%), Syncope (5%), Seizures (3%)
Epstein et al. (5)	Frequency and clinical spectrum of in-flight medical incidents during domestic and international flights	3,555	Flight diversions (*n* = 21): Cardiac (52%), Neurological (14%), Endocrine (10%), Respiratory (10%)
Gardelof (19)	In-flight medical emergencies. American and European viewpoints on the duties of health care personnel	NA	Total acute event (13%); Severe emergency: Cardiac (46%), Neurological (18%), Respiratory (6%)
Graf et al. (20)	Flight and altitude medicine for anesthetists-part 3: emergencies on board commercial aircraft	NA	Cardiovascular & Neurology/ Psychiatry (43%); Gastrointestinal (34%); Accidents (12%) (e.g., impact trauma; burns, scalds; cuts, fractures)
Hinkelbein et al. (13)	Emergencies in the sky: In-flight medical emergencies during commercial air transport	Review	Cardiac/Syncope (50.3%); Infectious disease (27%); neurological (23.4%). Most common mild problems: Nausea and vomiting.
Kim et al. (3)	Comparison of inflight first aid performed by cabin crew members and medical volunteers	2,818	*In-flight events requiring first aid*: Syncope or presyncope (18.1%), trauma (14.1%), Nausea or vomiting (10.1%), Respiratory (9.9%), Digestive (9.6%)
Kesapli et al. (4)	Inflight emergencies during Eurasian flights	1,312	Medical: Deterioration (23.7%), Shortness of breath (11.1%), Hypertension (6.3%), Nausea (5.6%), Abdominal pain (5%) Traumatic: Burns (16.8%), Soft tissue injuries (3.1%), Lacerations (0.2%).
Linthorst et al. (21)	Medical assistance by doctors on board an aircraft	NA	Most common medical problems: Vasovagal collapse, Dizziness, Gastro-intestinal, Cardiac complaints.
Martin-Gill et al. (6)	In-flight medical emergencies. A review.	49,100	Syncope/Near-Syncope (32.7%), Gastrointestinal (14.8%), Respiratory (10.1%), Cardiovascular (7.0%), Neurological (5.5%)
Makino et al. (22)	International airport and emergency medical care	2,696	(Airport Clinic) Acute abdomen (29.2%), Injuries (14.7%), Respiratory diseases (12.5%), Infectious diseases (7.5%), Ischemic heart diseases (6.4%)
Marsan et al. (23)	Outcomes of travelers who refuse transport after emergency medical services evaluation at an international airport	90	Trauma-related (34%), Neurologic (19%), Gastrointestinal (11%), Respiratory (8%), Psychiatric/intoxication-related (8%)
Meyer et al. (24)	Changes in heart rate and rhythm during a crossover study of simulated commercial flight in older and vulnerable participants	47	(In older, vulnerable participants) Heart failure (19.1%)
Pauline et al. (25)	Pediatric and adult emergencies on French airlines	581	Neurological: Syncope (40%), Seizures (4%); Gastrointestinal, metabolic disorders (20%); Respiratory- dyspnea (7%); Psychological- Anxiety (8%); Cardiovascular (6%)
Peterson et al. (7)	Outcomes of medical emergencies on commercial airline flights.	11,920	Syncope or presyncope (37.4%), Respiratory symptoms (12.1%), Nausea or vomiting (9.5%), Cardiac symptoms (7.7%), Seizures (5.8%)
Rotta et al. (11)	Characterization of in-flight medical events involving children on commercial airline flights	11,719	(In children) Nausea or vomiting (33.9%), Fever (22.2%), Acute allergic reaction (5.5%), Abdominal pain (4.7%), Gastroenteritis (4.5%)
Sand et al. (26)	Surgical and medical emergencies on board European aircraft: a retrospective study of 10,189 cases.	10,189	Most common medical emergencies: Syncope (53.5%), Gastrointestinal disorders (8.9%), cardiac conditions (4.9%). Most common surgical emergencies: thrombosis (0.5%), appendicitis (0.25%)

The rate of in-flight medical emergencies that lead to death prior to receiving medical assistance on the ground ranges from 0.3 to 0.67% (5, 7, 16, 25). The mortality rates were also low in reports that evaluated all medical events occurring on airport premises (14, 22). In the present study, none of the GBME cases were fatal (at least on-site), which is similar to other reports (5). Nevertheless, among the 19 critical cases that required flight diversion or re-entry, the most common medical emergency was neurological, representing nearly half (8/19) of cases; forced landings were also due to seizures, loss of consciousness and cardiovascular symptoms (palpitations, chest pain, etc.). Three patients treated on the ground were diagnosed as psychological-related panic causing hyperventilation in flight, and these patients were evaluated and discharged from emergency care. It has been reported that among cases evaluated, when physicians were available to participate in decision-making for flight diversion, related hospital admission rates were 49% compared to 15% when physicians were not onboard flights (27). However, we do not have data on availability of trained flight crews or presence of medical personnel, especially physicians.

Only one patient among the 19 IFME continued on the scheduled flight after medical examination for neurologic motion sickness and it was determined that hospitalization for further treatment was unnecessary. Among IFME passengers receiving treatment, the duration between emergency landing preparation and examination by ground-based medical staff was notably within 30 min. This is remarkable given that, in our study, the airport medical center data does not include information about passengers' in-flight condition or severity, and the evaluation process for making decisions to land are also not known. Therefore, the airport medical personnel must be prepared to evaluate patients upon landing. This may suggest a need for industry-wide standardized reporting and documentation of IFME so that the receiving medical personnel and facilities are able to better care for the patient. These cases suggest that all airport clinic staff may benefit from conducting regular “rehearsals” to improve the provision of rapid diagnosis and treatment in clinical emergencies. The time before an emergency medical team arrives is often the most critical in terms of saving lives, and thus it is also important to strengthen staff and public knowledge of and willingness to perform CPR as first aid in public places.

Of note, a high proportion of travelers in the present study were non-Taiwanese nationals and chose to continue their scheduled trips without treatment, so that subsequent follow-up was not feasible, and their final status remains unknown. Nonetheless, a prospective study evaluating short-term outcomes of flight passengers refusing transport after emergency medical evaluation at international airports found that most were well without sequelae (25).

A recent study collected information on pediatric patients who experienced an IFME that required physician evaluation (14). Those authors observed a higher frequency of medical events due to dermatologic causes in children compared with adults (21 vs. 3%), which agrees with findings of the present study. In our study, a higher proportion of cardiovascular-related medical emergencies occurred in older adult airline passengers compared to other age subgroups. Similarly, recent in-flight environment simulation studies demonstrated that older adults (>50 or 60 years) were more likely to experience alterations in heart rate, cardiac rhythm, and pulmonary artery pressure (23, 24). Despite the limited number of medical emergencies in pediatric and older adult passengers, related differences in the disease spectrum should be considered when evaluating airport medical situations. Passengers who feel unwell before a flight should be encouraged to seek outreach medical services before boarding, to help avoid IFME and to minimize associated medical risk (12).

### Limitations

This study has a few limitations, including that it was retrospective study. However, prospective study of airport emergency medical services may be difficult, given the complexity surrounding in-flight vs. on ground delivery of medical services. Data retrieved were often incomplete such as missing passenger medical history and lack of definitive diagnoses, and the uneven distribution of cases may skew statistical analysis when comparing between in-flight and on-ground events. The number of emergency medical events that occurred in the airport could be underestimated as patients that were able to self-present to the airport clinic for assessment were not included and only patients requiring out-reach medical service were analyzed in this study. In addition, there could be underestimation from GBME in the post-flight period for travelers who have left the airport and present to nearby medical centers outside of the airport.

Since 2020, the COVID-19 pandemic has undoubtedly changed air travel. Consequences of travel restriction imposed by governments worldwide to prevent the import of COVID-19 from outside of national borders, significantly impacted air travel (28). Compared to pre-COVID-19 period, the requirement of outreach service decreased as the number of passengers significantly decreased. However, as travelers were more aware of and alerted to COVID-19 infection risk, difference in ranking for most common chief complaints such as fever and respiratory related symptoms are expected. Data for 2020 and beyond warrants further analysis.

## Conclusions

TIA is particularly well-prepared to handle medical emergencies because of its location and existing medical resources, including the affiliated on-site medical center. The clinical spectrum of the most frequent emergency medical events that occur at TIA ranges from neurologic, gastrointestinal, respiratory, trauma to serious cardiovascular diagnoses requiring hospitalization. No significant differences are found between the types of IFME and GBME but the former, while fewer, represent more severe emergencies with poorer outcomes. Results of this study provide information to guide staff training and planning for the necessary medical supplies and procedures that will help meet the clinical demand in the setting of emergency medical outreach. The additional knowledge gained about age-associated medical events may help to improve in-flight and ground-based protocols in dealing with medical emergencies in these populations.

## Data Availability Statement

The original contributions presented in the study are included in the article/Supplementary Material, further inquiries can be directed to the corresponding author.

## Ethics Statement

The studies involving human participants were reviewed and approved by Landseed International Hospital. The ethics committee waived the requirement of written informed consent for participation.

## Author Contributions

S-TH and C-HL conceived and designed the study and performed the supervision. C-HL and Y-FS performed the acquisition of the data and draft the manuscript. S-TH did a critical revision of the manuscript. All authors analyzed and interpreted the data, conducted the analyses, performed the literature research, administrative, and technical material support, and provided the final approval of the manuscript.

## Conflict of Interest

S-TH was employed by Landseed Medical Clinic at Taiwan Taoyuan International Airport. The remaining authors declare that the research was conducted in the absence of any commercial or financial relationships that could be construed as a potential conflict of interest.

## Publisher's Note

All claims expressed in this article are solely those of the authors and do not necessarily represent those of their affiliated organizations, or those of the publisher, the editors and the reviewers. Any product that may be evaluated in this article, or claim that may be made by its manufacturer, is not guaranteed or endorsed by the publisher.

## Supplementary Material

Supplementary Table 1 Equipment and medications in the emergency medical kit.

Supplementary Table 2 Diagnoses between ground-based medical events and in-flight medical events by age.

Supplementary Figure 1 Overall disease symptoms by diagnosis and chief complaints.

Supplementary Figure 2 Disease symptoms of chief complaints between in-flight and ground-based medical events **(A)** and between in-flight medical events with diversion/or re-entry and scheduled landing **(B)**.
