# Supplementary material for: A Comparison of In-flight and Ground-Based Emergency Medical Events on the Clinical Demand for Outreach Medical Services at Taoyuan International Airport, Taiwan
Source: Front Public Health. 2021 Jul 23;9:663108. doi: 10.3389/fpubh.2021.663108 (PMC8342757; doi:10.3389/fpubh.2021.663108)
Supplement: Supplementary Table 1 — Equipment and medications in the emergency medical kit. [file Table_1.docx]

**Supplementary Table S1. Equipment and medications in the emergency medical kit.**

| Equipment | Medications | | | |
| --- | --- | --- | --- | --- |
|  | **Injectable** | **Ointment** | **Tablets** | **Tablets (continued)** |
| Laryngeal mask airway (LMA) 4#, 5# | Atropine | Kenalog x 1 | NTG bottle x 1 | KBT x 14 |
| Oropharyngeal airway(7.0FR/7.5FR/8.0FR) and Nasal airway (7.5#) | Aminophyllin x 2 | Eye (Tetracycline) x 1 | Actifed(peace) x 14 | Medicon-A x14 |
| Nasal cannula, Resuscitation mask, Bag valve mask | Xylocaine x 2 | Neomycin x 1 | Adalat-5 x 4 | Mgo x14 |
| Nasogastric tube(14Fr/16Fr) | Bosmin x 10 | Slivadene 20mg x 1 | Adalat-10 x 4 | Mucaine(Strocain) x14 |
| Triangle scarf | Dopamin x 2 | FOCUS x 1 | Allegra x 6 | Motilium x 14 |
| Protective gloves | Buscopan x 2 | Scheree x 1 | Amoxicillin-500 x 20 | Motilium (supp) x 4 |
| Stethoscope | Decadrone x 1 | Sinomin x 1 | Ativan x 10 | Novamin x 14 |
| Syringes(5ml,10ml) | Novamin x 2 | Emadine x 1 | Augmentin 1g x 14 | Norvasc x 5 |
| Needles (18-20-22- gauge) | Primperan x 1 | (Ear) Tarivid x 1 | Bansau x 14 | Panadol x 14 |
| Intravenous line administration set | Voren x 2 |  | Berotec x 14 | Predonine x 14 |
| Tape scissor | Vena x 2 |  | Bokey(Aspirin-100) x 8 | Periactin x 14 |
| Tourniquet | Lasix x 1 |  | Bonamin x 14 | Ponstan x 14 |
| Alcohol sponges | Dormicum x 1 |  | Buscopan x 14 | Primperan x 14 |
| Sphygmomanometer | Holdol x 1 |  | Buwecon x 12 | Pyridium x 14 |
| Glucose meter | Valium x 1 |  | Cafergot x 6 | Solaxin x 14 |
| Oximeter | Morphine x 1 |  | Cataflam(Voltaren-25) x 14 | Trandate x 4 |
| Pen light | Solu-medral 40mg x 1 |  | Cephadol x 14 | Transamin x 14 |
| Disposable towel drapes (sterile) | Solucortef x 1 |  | Clarinase x 8 | U-save-500 x 20 |
| AED (when required) |  |  | Cety x 5 | Valium-2(tube) x 10 |
| Oxygen (when required) |  |  | Capoten x 4 | Xanax(tube) x 8 |
| Portable EKG (when required) |  |  | Colchicine x 14 | Xanthium x 10 |
|  |  |  | Duspatalin x 14 | Voltaren-12.5 supp x 4 |
|  |  |  | Gascon x 14 |  |
|  |  |  | Imodium x 5 |  |
|  |  |  | Incidal x 14 |  |
|  |  |  | Inderal-10 x 14 |  |
