# Supplementary material for: A Comparison of In-flight and Ground-Based Emergency Medical Events on the Clinical Demand for Outreach Medical Services at Taoyuan International Airport, Taiwan
Source: Front Public Health. 2021 Jul 23;9:663108. doi: 10.3389/fpubh.2021.663108 (PMC8342757; doi:10.3389/fpubh.2021.663108)
Supplement: Supplementary Table 2 — Diagnoses between ground-based medical events and in-flight medical events by age. [file Table_2.DOCX]

**Supplementary Table S2. Diagnoses between ground-based medical events and in-flight medical events by age.**

|  | GBME (n=1227) | | |  | IFME (n=274) | | |  | IFME with  Diversion or re-entry (n=19) | | |  | IFME with  Scheduled landing  (n=255) | | |
| --- | --- | --- | --- | --- | --- | --- | --- | --- | --- | --- | --- | --- | --- | --- | --- |
| Diagnosis | <=18  yrs | 18-64  yrs | >=65  yrs |  | <=18  yrs | 18-64  yrs | >=65  yrs |  | <=18  yrs | 18-64 yrs | >=65 yrs |  | <=18 yrs | 18-64  yrs | >=65  yrs |
| Neurological | 29 (10.1) | 205 (71.7) | 52 (18.2) |  | 4 (7.2) | 39 (70.9) | 12 (21.8) |  | 1 (11.1) | 7 (77.8) | 1 (11.1) |  | 3 (6.5) | 32 (69.6) | 11 (23.9) |
| Gastrointestinal | 46 (17.1) | 199 (74) | 24 (8.9) |  | 5 (8.7) | 47 (81) | 6 (10.3) |  | 0 | 1 (100) | 0 |  | 5 (8.8) | 46 (80.7) | 6 (10.5) |
| Trauma | 41 (17.3) | 129 (54.4) | 67 (28.3) |  | 3 (6.2) | 36 (75) | 9 (18.8) |  | 0 | 1 (100) | 0 |  | 3 (6.4) | 35 (74.5) | 9 (19.1) |
| Respiratory | 50 (50) | 32 (32) | 18 (18) |  | 5 (16.7) | 19 (63.3) | 6 (20) |  | 1 (100) | 0 | 0 |  | 4 (13.8) | 19 (65.5) | 6 (20.7) |
| Cardiovascular | 0 | 57 (60.6) | 37 (39.4) |  | 0 | 18 (69.2) | 8 (30.8) |  | 0 | 2 (100) | 0 |  | 0 | 16 (66.7) | 8 (33.3) |
| Psychological | 1 (1.9) | 50 (94.3) | 2 (3.8) |  | 0 | 11 (100) | 0 |  | 0 | 3 (100) | 0 |  | 0 | 8 (100) | 0 |
| Ophthalmology/Ear Nose Throat | 7 (21.2) | 23 (69.7) | 3 (9.1) |  | 0 | 1 (33.3) | 2 (66.7) |  | 0 | 0 | 0 |  | 0 | 1 (33.3) | 2 (66.7) |
| Dermatology | 6 (22.2) | 20 (74.1) | 1 (3.7) |  | 4 (57.1) | 3 (42.9) | 0 |  | 0 | 0 | 0 |  | 4 (57.1) | 3 (42.9) | 0 |
| Genitourinary | 0 | 23 (88.5) | 3 (11.5) |  | 0 | 5 (71.4) | 2 (28.6) |  | 0 | 0 | 0 |  | 0 | 5 (71.4) | 2 (28.6) |
| Out-of-hospital cardiac arrest | 0 | 11 (55) | 9 (45) |  | 0 | 4 (44.4) | 5 (55.6) |  | 0 | 1 (50) | 1 (50) |  | 0 | 3 (42.9) | 4 (57.1) |
| Gynecologic | 1 (6.7) | 14 (93.3) | 0 |  | 0 | 10 (100) | 0 |  | 0 | 0 | 0 |  | 0 | 10 (100) | 0 |
| Musculoskeletal | 1 (5.6) | 11 (61.1) | 6 (33.3) |  | 0 | 1 (100) | 0 |  | 0 | 0 | 0 |  | 0 | 1 (100) | 0 |
| Infectious disease | 5 (31.2) | 7 (43.8) | 4 (25) |  | 0 | 2 (66.7) | 1 (33.3) |  | 0 | 0 | 0 |  | 0 | 2 (66.7) | 1 (33.3) |
| Alcohol/Drug | 0 | 17 (100) | 0 |  | 0 | 1 (100) | 0 |  | 0 | 0 | 0 |  | 0 | 1 (100) | 0 |
| Diabetes mellitus | 0 | 9 (81.8) | 2 (18.2) |  | 0 | 3 (60) | 2 (40) |  | 0 | 0 | 0 |  | 0 | 3 (60) | 2 (40) |
| Fever | 0 | 2 (66.7) | 1 (33.3) |  | 0 | 0 | 0 |  | 0 | 0 | 0 |  | 0 | 0 | 0 |
| Other | 0 | 2 (100) | 0 |  | 0 | 0 | 0 |  | 0 | 0 | 0 |  | 0 | 0 | 0 |

Data are summarized as n (%).

Abbreviations: GBME, ground-based medical events; IFME, in-flight medical events.
