# Supplementary figures and images for: A Comparison of In-flight and Ground-Based Emergency Medical Events on the Clinical Demand for Outreach Medical Services at Taoyuan International Airport, Taiwan
Source: Front Public Health. 2021 Jul 23;9:663108. doi: 10.3389/fpubh.2021.663108 (PMC8342757; doi:10.3389/fpubh.2021.663108)

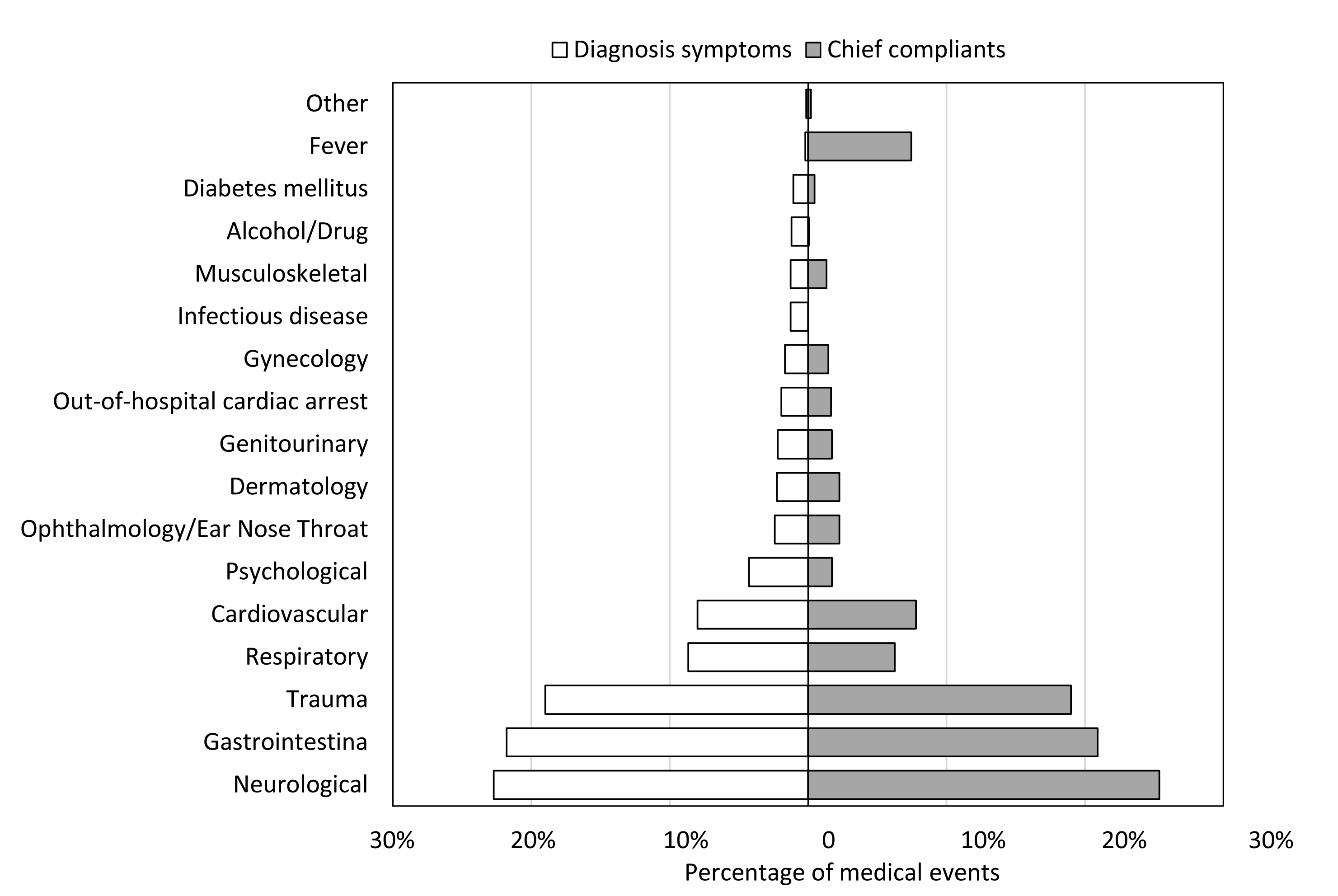

Supplement: Supplementary Figure 1 — Overall disease symptoms by diagnosis and chief complaints. [file Image_1.tif]

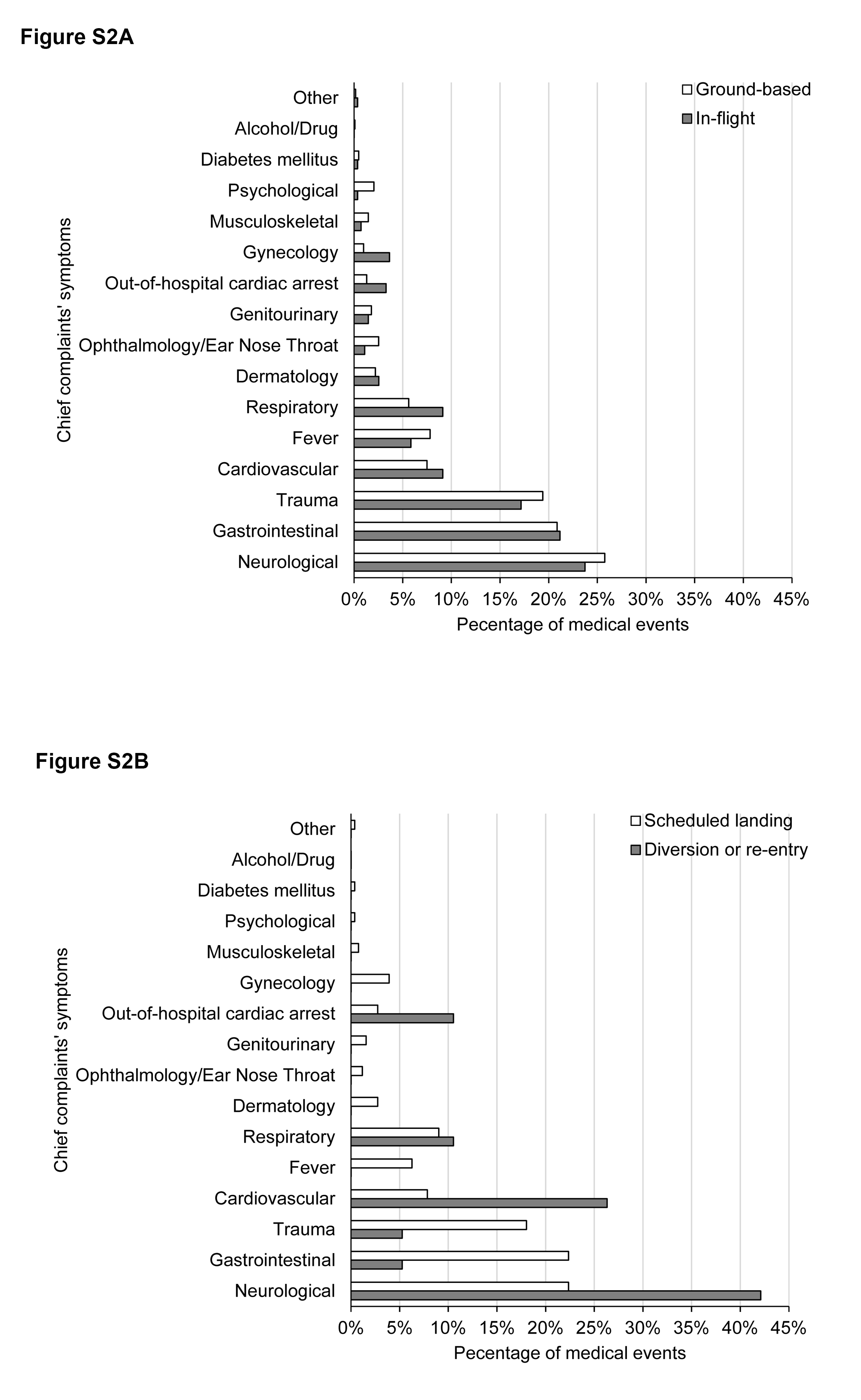

Supplement: Supplementary Figure 2 — Disease symptoms of chief complaints between in-flight and ground-based medical events (A) and between in-flight medical events with diversion/or re-entry and scheduled landing (B). [file Image_2.tif]
